# The virulence toolkit of *Staphylococcus aureus*: a comprehensive review of toxin diversity, molecular mechanisms, and clinical implications

**DOI:** 10.1007/s10096-025-05148-y

**Published:** 2025-05-05

**Authors:** Stefano Di Bella, Bruna Marini, Giacomo Stroffolini, Nicholas Geremia, Daniele Roberto Giacobbe, Floriana Campanile, Michele Bartoletti, Giulia Alloisio, Ludovica Di Risio, Giulia Viglietti, Luigi Principe, Venera Costantino, Marina Busetti, Verena Zerbato, Filippo Mearelli, Gianni Biolo, Alessio Nunnari, Claudia Maria Cafiero, Alessandra di Masi

**Affiliations:** 1 Clinical Department of Medical, Surgical, and Health Sciences, Trieste University, Strada di Fiume, 477, Trieste, 34129 Italy; 2 Infectious Diseases Unit, Trieste University Hospital (ASUGI), Trieste, Italy; 3 Ulisse BioMed, Area Science Park, Trieste, Italy; 4 Department of Infectious-Tropical Diseases and Microbiology, IRCCS Sacro Cuore Don Calabria Hospital, Negrar, Verona, Italy; 5 Infectious Diseases Service, Lausanne University Hospital, Lausanne, Switzerland; 6 Unit of Infectious Diseases, Department of Clinical Medicine, Ospedale “dell’Angelo”, Venice, Italy; 7 Unit of Infectious Diseases, Department of Clinical Medicine, Ospedale Civile “S.S. Giovanni e Paolo”, Venice, Italy; 8 UO Clinica Malattie Infettive, IRCCS Ospedale Policlinico San Martino, Genoa, Italy; 9 Department of Health Sciences (DISSAL), University of Genoa, Genoa, Italy; 10 Department of Biomedical and Biotechnological Sciences, Section of Microbiology, University of Catania, Catania, Italy; 11 Department of Biomedical Sciences, Humanitas University, Pieve Emanuele, Milan, Italy; 12 IRCCS Humanitas Research Hospital, Milan, Rozzano Italy; 13 Department of Sciences, Roma Tre University, Roma, Italy; 14 Microbiology and Virology Unit, Great Metropolitan Hospital “Bianchi-Melacrino-Morelli”, Reggio Calabria, Italy; 15 Microbiology Unit, Trieste University Hospital (ASUGI), Trieste, Italy; 16 Clinica Medica, Trieste University Hospital (ASUGI), Trieste, Italy

**Keywords:** *Staphylococcus aureus*, Toxins, Antibiotic therapy, Monoclonal antibodies, Anti-inflammatory treatments

## Abstract

**Purpose:**

This review examines the pathogenic mechanisms of *Staphylococcus aureus*, emphasizing its toxin-driven virulence factors, including pore-forming toxins, exfoliative toxins, and superantigens.

**Methods:**

This paper was conducted using the available literature (PubMed/MEDLINE/Google Scholar and books written by experts in pharmacology and infectious diseases).

**Results:**

Toxins are crucial in promoting tissue invasion, immune system evasion, and the development of systemic diseases. Notably, the qualitative and quantitative expression of these toxins influences the clinical presentation and severity of *S. aureus* infections. The paper explores toxins’ role in *S. aureus* pathogenesis and clinical manifestations as well as current and emerging therapeutic strategies aimed at targeting these toxins, including antibiotics, monoclonal antibodies, and anti-inflammatory treatments. Additionally, it highlights the potential of novel inhibitors and vaccines to neutralize specific toxins and prevent toxin-mediated diseases.

**Conclusion:**

By combining antimicrobial therapies with approaches that neutralize toxins and modulate the immune response, clinicians can improve outcomes in patients affected by *S. aureus* infections.

## Introduction

*Staphylococcus aureus* is a Gram-positive, facultative aerobic bacterium. As a common commensal in humans, this microorganism is present on nasal mucosa and skin of 30% of healthy people [1].

Pathogenic infections caused by *S. aureus* vary widely, ranging from mild skin infections to severe necrotizing pneumonia and septic shock. *S. aureus* is also a leading cause of bacteremia and infective endocarditis and can also result in osteoarticular, skin and soft tissue, pleuropulmonary, and device-related infections. Notably, *S. aureus* remains the most frequent causative agent of hospital-acquired infections, with mortality rates as high as 20% in bloodstream infections [2]. The incidence of *S. aureus* infection is increasing [3], posing a significant public health challenge. Despite advancements in antibiotic treatments and strategies, mortality rates for *S. aureus* bloodstream infections have remained unchanged since the 1990s, highlighting the need for therapies that target the bacterium and inhibit the production and effects of its toxins.

For this reason, monoclonal antibodies targeting specific virulence factors have emerged as a promising strategy for tackling these infections [4].

In pathological conditions, *S. aureus* produces diverse virulence factors that enable it to evade the host immune system and establish infections. Among these, secreted toxins play a pivotal role, exhibiting specificity for various cell types and distinct mechanisms of action. These toxins demonstrate functional redundancy, which may contribute to *S. aureus* ability to withstand different therapeutic interventions [5]. By targeting structural tissues and immune cells, toxins synergistically enhance *S. aureus* virulence, enabling the bacterium to cause infections ranging from localized skin conditions to life-threatening systemic diseases.

This review aims to provide a comprehensive overview of the virulence factors produced by *S. aureus* in pathological contexts, their clinical counterpart and the therapeutic strategies developed to target them.

## *Staphylococcus aureus* toxins

Toxins produced by *S. aureus* work synergistically to enhance its ability to infect and damage the host. These toxins target various aspects of the host’s immune and structural defenses, allowing the bacterium to establish, maintain, and spread infections. In particular, they support the pathogen in achieving three main effects [5]. Promote tissue invasion by disruption of cell-cell adhesion in the skin or by cell lysis induction; this opens pathways for the bacteria to invade deeper tissues and achieve further breakdown of tissue barriers [5, 6]. Facilitate the evasion of immune defenses. Diverse toxins target and kill different immune cells, including neutrophils and macrophages, by disrupting their membranes [5]. This weakens the immune response and allows the bacteria to persist in the host.

Additionally, toxins such as superantigens can hyperactivate T cells, leading to an overwhelming cytokine storm that can suppress effective immune responses and cause systemic damage [5, 6]. Finally, the toxins’ combined effects result in a spectrum of diseases. Superficial skin infections, such as impetigo and abscesses, are caused by localized damage from cytotoxins and immune evasion mechanisms. Systemic illnesses like staphylococcal scalded skin syndrome (SSSS) or toxic shock syndrome (TSS) result from the systemic dissemination of exfoliative toxins or superantigens, causing widespread tissue damage, immune dysregulation and, in severe cases, multi-organ failure [5, 7].

A deeper understanding of the production mechanisms and the role of these virulence factors is urgently needed, as well as the interactions between the pathogen and the host, which play an important role in the disease’s onset. The main *S. aureus* toxins, whose key characteristics are summarized in Table 1, can be divided into three major groups: (i) Pore-Forming Toxins (PFTs), (ii) Exfoliative toxins (ETs) and (iii) Superantigens (SAgs) [8].

**Table 1:** The main characteristics of *S. aureus* toxins are strain diffusion and clinical implications

Class	Category		Genomic location	% strains	Mechanism	Cellular receptors	Target cells	Disease	Disease
Pore-Forming Toxins	α-hemolysins		Core genome	95%	β-barrel channel	ADAM10; disintegrin	Erythrocytes; platelets; endothelial cells; B cells; T cells	Epithelium degradation; bacterial dissemination; ion loss; cell death	Skin and soft tissue infections, sepsis, bacteraemia, osteomyelitis, bone infections
	β-hemolysins		Chromosome and mobile genetic elements	80–90%		Sphingomyelin	Epithelial cells; leukocytes; erythrocytes; keratinocytes		Skin and soft tissue infections, device-related infections, endocarditis
	Leukotoxins	PVL	Temperate bacteriophages	36%		C5aR	Monocytes; neutrophils; macrophages	Ca²⁺ influx; cell death; tissue necrosis	Severe necrotizing pneumonia
		γ-hemolysin	Core genome	99%		C5aR; CXCR1; CXCR2	Erythrocytes	Cell lysis	Septic arthritis; sepsis; bacteraemia; endocarditis
		LukAB/LukGH		~ 100%		CD11b	Polymorphonuclear cells; neutrophils	Immune evasion	Osteomyelitis and bone infections
		LukED		70–80%		CCR5; CXCR1; CXCR2	Leukocytes; T cells; monocytes; endothelial cells		SSTIs, necrotizing pneumonia, bacteraemia, endocarditis
	Phenol-soluble modulins			~ 100%	α-helices	Non-specific	Leukocytes; erythrocytes	Membrane disruption; biofilm induction; inflammation; immune	Skin and soft tissue infections
Exfoliative toxins			Core genome and temperate bacteriophage	5–20%	Proteolysis	Desmoglein-1	Keratinocytes	Skin disruption	Staphylococcal scalded skin syndrome; bullous impetigo; dermatitis
Superantigens	TSST-1		Mobile genetic elements	20–50%	T-cell overactivation	TCR; MHC II molecules	Antigen-presenting cells	T-cell overactivation and death; cytokine storm	Toxic shock syndrome (TSS); systemic inflammation
	Staphylococcal enterotoxins			10–50%				GI symptoms: nausea, vomiting, diarrhea	Food poisoning
	Enterotoxin-like proteins			80–90%				Immunomodulatory effects; virulence enhancement	Not directly associated with pathologies

### Pore-forming toxins (PFTs)

**Fig. 1:**
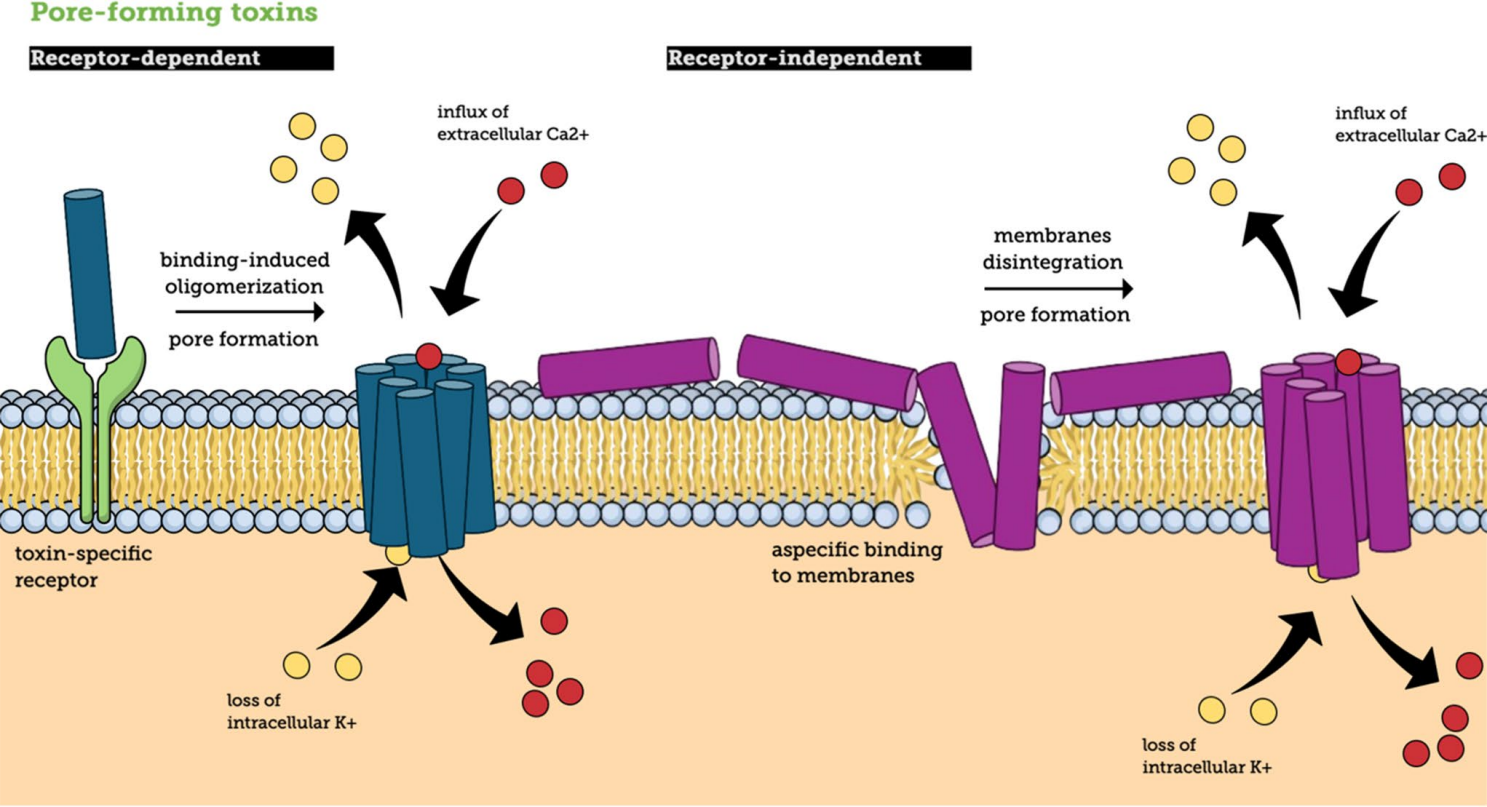
General principles of pore-forming toxins (PFT) mechanism. Pore-forming toxins interact with cell membranes through specific binding to a membrane receptor or direct interaction with membrane components. Depending on the toxin class, the binding mechanism differs. Upon binding, the toxins oligomerize to form pores in the membrane, leading to dysregulated ion fluxes that ultimately results in cell death

PFTs are protein toxins produced by various bacteria, including *S. aureus*, that disrupt host cell membranes by creating pores. These pores compromise the integrity of the cell membrane, leading to the leakage of ions and molecules, which ultimately causes cell death (Fig. 1) [5]. PFTs play a critical role in the virulence of many pathogens, aiding in tissue invasion and immune evasion. PFTs can be further divided into four types: α-hemolysins (Hla or α-toxin), β-hemolysins (Hlb), leukotoxins and phenol-soluble modulins (PSMs) [5].

#### α-hemolysins or α-toxins

α-hemolysins (or α-toxins, α hemolysins) were the first described PFTs encoded by 95% of *S. aureus* strains, localized on the core genome. α-hemolysin is a water-soluble monomer of 33 kDa, which, after the attachment to its ligand, can assemble into a beta-barrel homo-heptameric pre-pore. These domains allow the formation of a beta-barrel channel on the target cell’s surface [5].

The transmembrane disintegrin and metalloproteinase containing protein ADAM10 is the receptor for α-hemolysin, and this explains the specificity of action shown by the toxin (α-hemolysin mediated lysis occurs in erythrocytes, platelets, endothelial cells and human B and T cells) [9]. Once bound by α-hemolysin, ADAM 10 is activated and degrades E-cadherin, resulting in a loss of homotypic interactions between cadherin molecules of adjacent cells at adherent junctions. This event leads to the impairment of the structure of epithelium and promotes the dissemination of *S. aureus* [8]. A pore on the host cell’s surface allows the loss of intracellular K^+^ and the influx of extracellular Ca^2+^, leading to cell death [10].

Probably after bacterial death, circulating α-hemolysin released could activate the sphingomyelinase/ceramide system [11], which leads to the degradation of tight junctions of endothelial cells in the lung, causing severe pulmonary edema [12]. This mechanism may explain why patients develop pulmonary edema even with proper antibiotic treatments. Moreover, activation of the sphingomyelinase/ceramide system is also linked to the activation of the inflammasome, resulting in the release of IL-1β and TNF-α [13]. As part of the core genome, this hemolysin is broadly expressed and plays a central role in the virulence mechanisms of S. aureus infections, prominently involved in most syndromic manifestations. While clinical data on the role of α-hemolysin in bacteremia pathogenesis remain limited, in vitro studies have shown that α-hemolysin activity in methicillin-resistant *S. aureus* (MRSA) strains isolated from bacteremia patients is significantly associated with an increased risk of thrombocytopenia and mortality. However, these findings are based on a limited sample size, underscoring the need for further research to better define its specific role across different clinical contexts and syndromes [14].

#### β-hemolysins (Hlb or Sphingomyelinase C)

β-hemolysins (or Hlb) belong to the sphingomyelinase family, enzymes deleting sphingomyelin, the most abundant sphingolipid in the eukaryotic membrane [15, 16]. Most genes encode these enzymes on the bacterial chromosome, although mobile genetic elements carry some [15, 16]. β-hemolysin is a β-toxin that contributes to *S.aureus* virulence by exerting a cytolytic effect on various host cells, including epithelial cells, leukocytes, and red blood cells. Its activity destabilizes cell membranes, causing cell lysis and releasing intracellular contents, thereby promoting the bacterium’s spread within infected tissues. β-hemolysin production by *S. aureus* damages keratinocytes, leading to skin colonization [17].

Additionally, β-hemolysin modulates the host immune response, aiding in immune evasion and increasing the severity of *S. aureus* infections, such as abscesses, skin infections and, in more severe cases, sepsis. Like α-hemolysin, β-hemolysin is constitutively expressed by *S. aureus*, making its clinical impact inherent to the bacteria. However, few studies have specifically evaluated the diversity of clinical outcomes associated with β-hemolysin activity. β-hemolysin might play a role in helping *S. aureus* colonize the skin. Moreover, altered β-hemolysin expression may influence the severity of *S. aureus*-related lung injury. Both experiences come from murine studies and need confirmation in humans [17, 18]. This limited research is primarily attributed to technical challenges inherent in laboratory techniques, which make it difficult to accurately measure and analyze the effects of β-hemolysin across different clinical scenarios.

#### Leukotoxins

Leukotoxins are a group of PFTs that specifically target and destroy leukocytes, playing a significant role in the microorganism’s ability to evade the host immune system and promote infection [19]. These toxins are bi-component proteins consisting of two distinct subunits that work together to form β-barrel pores in the membranes of target cells [20, 21]. The two subunits are classified as “S” (specificity) and “F” (forming) components. The S-component determines cell-type specificity by binding to specific cellular receptors. At the same time, the F-component is essential in dimerization and facilitates the formation of β-barrel pores that disrupt the cell membrane [8]. This process ultimately leads to cell lysis and the release of intracellular contents, causing inflammation and immune dysregulation [20, 21].

However, it has become increasingly evident that leukotoxins also act as potent activators of innate immunity. This dual role arises from their interactions with host cells and the inflammatory pathways they trigger. When leukotoxins bind to their specific receptors and form pores, they cause cell death and release danger-associated molecular patterns and pro-inflammatory cytokines from the lysed cells. These signals alert the immune system to infection, leading to the recruitment and activation of additional immune cells [22, 23]. Furthermore, sublytic concentrations of leukotoxins, which do not kill cells outright, can stimulate intracellular signaling pathways in immune cells. This can produce cytokines, chemokines, and other mediators that amplify the innate immune response. These effects demonstrate that leukotoxins play a complex role in modulating the immune response, acting as immune evasion tools and activators of inflammatory processes that shape the host-pathogen interaction [5].

So far, four bi-component leukotoxins have been identified from *S. aureus* strains causing infections in humans: Panton-Valentine Leukocidin (PVL), γ-hemolysins (Hlg), Leukotoxin ED (LukED), and Leukotoxin AB/GH (LukAB/LukGH). These toxins contribute to the severity of *S. aureus* infections as abscesses, necrotizing pneumonia and sepsis, by neutralizing immune defenses and enhancing bacterial survival and dissemination [19].

##### Panton-valentine leukocidin (PVL)

PVL, first observed in 1894 by Van der Velde and later isolated by Panton and Valentine in 1932, plays a key role in bacterial virulence. It consists of two subunits, LukS-PV and LukF-PV, which were purified in 1960 by Woodin and characterized by the different elution time in the chromatographic column in 38 kDa S (slow) and 32 kDa F (fast) subunits. These subunits interact on polymorphonuclear leukocyte membranes to form a β-barrel pore, a process influenced by environmental factors like calcium concentration [24]. In more detail, once bound on the host cell’s surface, LukS-PVL oligomerizes with LukF-PVL, forming a heterodimer. This heterodimer oligomerizes into heterotetramers by alternating LukS and LukF subunits, and subsequently, two heterotetramers can assemble into an octameric structure. This ring-shaped structure cannot be inserted inside the membrane until conformational changes occur that allow it to be inserted into the membrane, leading to cell death [20]. PVL targets monocytes, neutrophils, and macrophages, but not lymphocytes, and demonstrates species specificity, being highly toxic to human and rabbit cells but ineffective against murine cells, making the mouse model useless. However, the humanized mice model has been investigated for studying implant-associated osteomyelitis with community-acquired methicillin-resistant *S. aureus* (CA-MRSA) [25].

PVL mechanism involves binding to the C5a receptor on polymorphonuclear leukocytes, leading to pore formation and calcium influx, which activates polymorphonuclear leukocytes and triggers the release of granules [26]. This activation contributes to tissue necrosis observed in PVL-positive *S. aureus* lesions. Unlike α-hemolysins, encoded in the core genome, the *pvl* locus is carried by temperate bacteriophages, such as ɸSa2USA and ɸPVL, which integrate into the bacterial genome during lysogeny [27].

Data from 2004 to 2006 show that 36% of *S. aureus* isolates carried PVL genes, predominantly in MRSA strains, with most belonging to clonal complex 8 [28]. The sequence of PVL genes is conserved, with nonsynonymous R/H variations (arginine or histidine at amino acid 176). Research suggests that USA300 MRSA (CA-MRSA) strains from clonal complex 8 likely lost the *mecA* gene over time while retaining PVL genes, as *mecA* imposes a fitness cost in non-antibiotic environments. This highlights a selective advantage for PVL-positive strains in community settings [20, 28].

The integration of PVL-encoding phages into *S. aureus* genomes occurs at specific conserved sites, differing among clonal complexes. This lineage-phage specificity and horizontal gene transfer via phage transduction [29, 30] explain the distribution and expression variability of PVL genes across *S. aureus* strains. These findings underscore PVL’s importance in the evolution and pathogenicity of *S. aureus* strains.

As previously stated, CA-MRSA often exhibits greater fitness and virulence than its nosocomial counterparts. Outpatient clinics serve as the primary entry point for CA-MRSA into hospital settings. PVL is a key virulence factor, particularly in the skin and soft tissue infections (SSTIs), the most common symptom, lung infections (e.g., necrotizing pneumonia due to PVL-producing *S. aureus*) or other non-dermatological manifestations. *S. aureus* strains producing PVL (PVL-SA) are commonly linked to large, recurring abscesses in otherwise healthy young individuals. The rising prevalence of PVL-producing MRSA and the introduction of imported strains are reshaping the local MRSA landscape, which is heavily influenced by travel habits. Other risk factors appear to be close contact, contaminated items, crowding, cleanliness, cuts, and other compromised skin integrity. Notably, geographic variations in SSTI risk among travelers highlight the global heterogeneity in the distribution of virulent *S. aureus*. SSTIs caused by PVL-SA are more likely to recur than those caused by PVL-negative strains. Reports indicate that complicated SSTIs in travelers returning from non-temperate regions are more frequently associated with PVL-SA, contributing to the emergence and dissemination of virulent, antibiotic-resistant strains [31–34]. For what pertains to other non-dermatological conditions, severe musculoskeletal infections such as osteomyelitis, pyomyositis and septic arthritis, especially in children, have been described. From a diagnostic perspective, on top of relevant diagnostic samples, a specific molecular biology investigation should be demanded upon clinical suspicion, which is not routinely performed and frequently sent to national reference laboratories [35].

##### γ-hemolysin (Hlg)

The γ-hemolysins gene cluster encodes for three genes, coding for two class S proteins (HglA and HlgC) and one class F protein (HlgB) [36, 37]. The γ-toxin locus enables the formation of two functional S + F protein pairs: HlgA + HlgB and HlgC + HlgB. This locus is part of the core genome and is found in 99% of *S. aureus* strains. *hlg*B and *hlg*C are 98.5 and 99.1% identical to the *luk*F and *luk*S genes, respectively, encoding the F and S components of the PVL [38]. γ-hemolysins are associated with septic arthritis and weight loss in mice [39] as well as endophthalmitis in rabbits [40]; in mammals, they trigger the lysis of erythrocytes [37]. In a subsequent unrelated study, absolute quantification of HlgCB by mass spectrometry in community-acquired pneumonia isolates revealed significant variation in the levels of HlgC and HlgB between different isolates. Notable differences in HlgCB quantities were observed among clinical isolates from community-acquired pneumonia patients, and biomolecular analyses identified several SNPs in the promoter regions and a single nucleotide polymorphism in the 5’ UTR of *hlg*CB mRNA. These genetic variations lead to differential expression of *hlgCB*, which greatly affects the translation of *hlgC* mRNA and, consequently, the virulence of *S. aureus*. This type of analysis highlights the complexity of virulence factor expression in clinical strains [41].

##### LukAB/LukGH

LukAB, also known as LukGH, is a bicomponent, beta-barrel toxin produced by *S. aureus*; its role in evading human immune response has been mainly characterized in USA300 MRSA strain [42]. The success of this clone resides in its ability to escape the destruction by human polymorphonuclear leukocytes, a fundamental step in the immune response to *S. aureus* infection. This is achieved through LukAB, which is interestingly both a secreted protein in the extracellular medium and one of the most abundant surface-associated proteins of USA300 during its exponential growth [43, 44]. LukAB facilitates immune evasion by forming pores in the membranes of polymorphonuclear leukocytes, leading to their lysis. This toxin also enables *S. aureus* to escape from within neutrophils after being engulfed, which promotes bacterial survival and replication. The expression of LukAB is upregulated when *S. aureus* encounters neutrophils, and the growth medium and bacterial growth phase influence its production. Research findings indicate that strains lacking LukAB are less effective at evading neutrophil defenses and exhibit reduced virulence and growth rebound after phagocytosis. These insights highlight the critical role of LukAB in the pathogenicity of the USA300 strain, suggesting that it is a key factor in the success of *S. aureus* as a pathogen. Additionally, it should be highlighted that two-component systems are highly conserved across bacterial species and play a crucial role in enabling rapid sensing and response to changes in environmental conditions. *S. aureus* utilizes the *S. aureus* exoprotein expression (SaeRS) two-component system to detect host signals and initiate the transcription of virulence factors essential for pathogenesis. Some specific components of this system are critical for the proper transcription of *sae* target genes, such as *lukAB/lukGH*, which contribute to polymorphonuclear leukocyte lysis. Notably, these are among the most abundant surface-associated proteins in the USA300 strain, potentially playing a significant role in its virulence [44, 45].

##### LukED

LukED is a leukotoxin produced by *S. aureus*, which targets and kills leukocytes. It is a bicomponent pore-forming toxin consisting of two separate protein subunits that work together to form pores in the membranes of target cells. LukED specifically binds to chemokine receptors, with CCR5 being the first to be identified [46]. CCR5 is necessary and sufficient for killing T cells, macrophages, and dendritic cells, which are crucial for resolving infections. The *lukED* gene is present in most clinically relevant strains, particularly those associated with severe infections [47], that by producing high levels of LukED exploit the toxin to eliminate antigen-presenting cells and protective CCR5 + Th1/Th17 cells, impairing the host immune response. Other receptors LukED targets are CXCR1 and CXCR2, which are found on the surface of certain immune cells [48]. CXCR1 is predominantly found on neutrophils but is also present on monocytes, natural killer cells, some subsets of T lymphocytes, and epithelial cells. It plays a key role in neutrophil migration and activation in response to chemokines such as IL-8. Similarly, CXCR2 is expressed on neutrophils and shares significant functional overlap with CXCR1, binding to IL-8 and other chemokines like growth-related oncogene alpha. In addition to neutrophils, CXCR2 is found on monocytes, endothelial cells, basophils, and some epithelial cells, which contribute to the recruitment of immune cells to sites of inflammation and tissue injury. These receptors are critical for mediating immune cell migration and inflammatory responses. By disrupting these cells, LukED impairs the immune system’s ability to respond effectively to infection, allowing *S. aureus* to evade host defenses and promote its proliferation [48].

#### Phenol-soluble modulins (PSMs)

PSMs are a family of amphipathic peptides with a characteristic ⍺-helical structure encoded by staphylococci; they recently received attention due to their contribution to inflammatory response and structural integrity of robust *S. aureus* biofilms [49, 50].

They are encoded in different genomic locations and include α-type and β-type PSM, as well as δ-type PSM (or δ-toxin), which differ in charge and structural characteristics [50, 51]. These peptides form amphipathic α-helices that can interact with cytoplasmic membranes in a non-specific way (differently from other PFTs) [49, 50], causing membrane disintegration, with their effects influenced by the target cell membrane composition and charge [52]. In particular, PSM⍺-3 has shown the most potent activity against human leukocytes and erythrocytes at micromolar concentration [50].

PSMs play a key role in pathogenesis, contributing to biofilm structuring, surface spreading, and immune evasion by lysing neutrophils and inducing inflammation. Studies show that α-type PSMs are particularly important for the virulence of CA-MRSA, while δ-toxin and β-type PSMs have a limited role in infections. Indeed, PSMs have been seen to contribute to biofilm formation, a crucial step in staphylococcal colonization [50]. Biofilm further enhances bacterial survival and resistance to antibiotics and immune defenses, especially in MRSA [53].

The important role played by PSMs in the pathogenesis of *S. aureus* makes these peptides logical targets for therapies against highly virulent *S. aureus*. On top of immunomodulation properties, cytolysis, and antimicrobial properties leading to pathogen interference, it has been shown that PSMα peptides of *S. aureus* play a crucial role in enabling virulent *S. aureus* to cause skin infections and bacteremia in animal models. Additionally, all *S. aureus* PSM peptides promote the spread of biofilm-associated infections to other organs in the human body. These observations suggest that PSM peptides significantly influence the pathogenesis of major staphylococcal diseases and have evolved divergently to perform specialized functions in pathogenesis [51].

#### Production of PFTs in various *S. aureus* strains

Overall, the production of PFTs varies significantly among *S. aureus* strains, with notable differences between methicillin-susceptible *S. aureus* (MSSA) and MRSA strains, as well as between community- and hospital-associated isolates. Highly virulent strains, such as USA300, are often linked to severe infections due to the overproduction of toxins like PVL and α-toxin. The *S. aureus* strains producing PFTs are distributed across different clonal lineages. They can be categorized based on their origin (community or hospital-acquired), antibiotic resistance, and the presence of specific virulence genes. MSSA strains are typically associated with infections in the community and hospitals. These strains commonly produce α-hemolysin and γ-hemolysins, causing diseases such as SSTIs, pneumonia, and bloodstream infections [54].

On the other hand, MRSA strains are divided into two main groups. CA-MRSA strains are linked to infections in healthy individuals outside healthcare settings [55]. These strains are highly virulent, often producing toxins like PVL, which is associated with necrotizing or other severe skin infections; they also frequently express α-hemolysin. Examples include the USA300 clone in North America, a dominant PVL-producing strain, and ST80 in Europe [56]. Conversely, hospital-associated MRSA (HA-MRSA) strains predominantly cause infections in healthcare environments, often targeting immunocompromised patients or those with medical devices. However, the occurrence also depends on implementing infection prevention and control measures within the hospital setting. These strains often exhibit higher levels of antibiotic resistance and lower PVL production compared to CA-MRSA. However, they commonly produce α-hemolysin, γ-hemolysins, and LukED. Significant clones in this category include ST239, which has a global presence, and ST22 (EMRSA-15), dominant in Europe [57–59]. Genetic regulators influence the production of these toxins, such as the *agr* system (Accessory Gene Regulator), which modulates toxin expression in response to environmental signals [60].

### Exfoliative toxins (ETs)

**Fig. 2:**
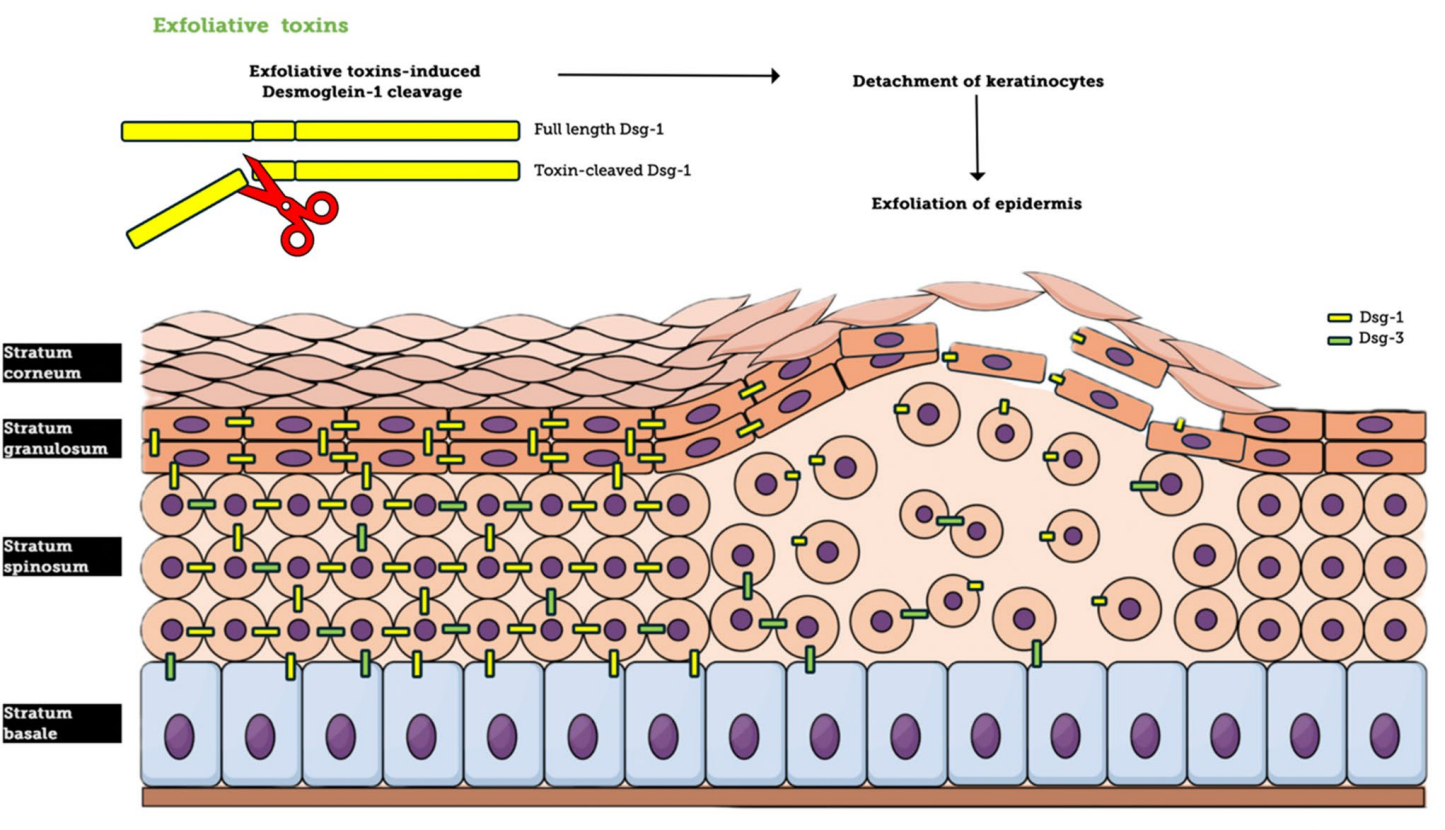
General principles of Exfoliative Toxins (ET) mechanism. ET specifically cleaves desmoglein-1, a critical component of cell junctions, leading to cell detachment and the exfoliation of epithelial tissue

ETs are proteolytic toxins produced by *S. aureus* that disrupt the skin’s epidermal layers, leading to conditions such as SSSS prevalently in newborns and immunocompromised adults [61, 62]. If treated adequately, the mortality rate in children is relatively low (< 5%); however, in adults, it rises to 59% [63]. ETs are serine proteases targeting desmoglein-1, a protein critical for cell-cell adhesion in the skin’s superficial layers. By cleaving desmoglein-1, these toxins cause keratinocyte detachment, leading to epidermal exfoliation [64] (Fig. 2).

ETs are typically produced by specific strains of *S. aureus* and are implicated in localized and systemic infections, particularly in infants, very low birth weight neonates and immunocompromised individuals [65]. These toxins are also heat-stable, allowing them to maintain activity in various conditions. Five types of exfoliative toxins (ETs) have been identified: exfoliative toxin A (ETA), exfoliative toxin B (ETB), exfoliative toxin C (ETC), exfoliative toxin D (ETD), and exfoliative toxin E (ETE). These toxins’ primary amino acid sequences consist of 242–247 amino acid residues in their mature form. The sequence identity among ETA, ETB, ETD, and ETE ranges from 43 to 63%; however, ETC does not exhibit any significant sequence identity with these toxins. ETC, also known as adenylosuccinate lyase, is not toxic to humans, and its three-dimensional (3D) structure differs from that of ETA, ETB, and ETE [66]. There are two major types: ETA, encoded by the chromosomally located *eta* gene, and ETB encoded by the plasmidic *etb* gene; the *eta* gene is horizontally acquired by the temperate bacteriophage (qETA) [67]. Notably, *eta* containing strains are associated with a recent rise in the occurrence of SSSS in the United States that has been documented [68] where SSSS cases rate at a pediatric hospital increased from 2.3 per 10,000 admissions in 2008 to 52.6 per 10,000 admissions in 2017 [69]. Most of such SSSS-associated infections were found to be sustained by MSSA [70], implying that such susceptible strains exploit virulence factors to cause significant morbidity and mortality. It must be noted that MSSA accounts for 59.7% of hospital-acquired infections and 60.1% of deaths [54]. Various reports have explored these toxin-associated infections. One study documented previously unidentified types of *etb*-positive plasmids isolated from impetigo strains linked to outbreaks of pemphigus neonatorum in Czech maternity hospitals [70]. Another research group report revealed significant genomic diversity among impetigo strains and the distribution of major genotypes in maternity hospitals in the Czech Republic and Slovakia [71]. Additionally, SSSS has been associated with severe prothrombotic conditions, a rare and potentially under-reported occurrence [72]. Clinicians should also note that SSSS can develop after burn injuries, particularly in infants [73].

In a British study investigating two SSSS outbreaks in a maternity unit, 500 pregnant women attending an antenatal clinic were screened for *S. aureus*-producing epidermolytic toxin. Although the prevalence was low (approximately 3%), the findings offer baseline data on *S. aureus* in the community. Identifying methicillin-resistant and toxin-producing strains highlights a potential reservoir for outbreaks, emphasizing the importance of management strategies, especially upon hospital admission of such carriers [74]. Importantly, from an infection control perspective, contact measures may be relevant in some cases [75].

#### Production of exfoliative toxins in various *S. aureus* strains

The distribution of ETs in *S. aureus* varies among strains and is closely linked to each strain’s genetic makeup and epidemiological context. Exfoliative toxins, primarily ETA and ETB, are typically associated with specific clonal lineages of *S. aureus* prevalent in community and hospital settings. ETA is found in strains more commonly associated with community-acquired infections [76]. It has a broader distribution among MSSA strains and is frequently linked to strains isolated from pediatric infections, particularly in localized or systemic SSSS cases. On the other hand, ETB is often carried by specific strains that exhibit a higher virulence potential, particularly in neonatal and pediatric cases of SSSS. ETB is less widespread than ETA but is significant in specific geographic and epidemiological contexts, where plasmid transmission plays a role in its dissemination [68]. The distribution of these toxins also highlights the role of mobile genetic elements, such as plasmids and pathogenicity islands, in spreading ET-encoding genes across strains, allowing *S. aureus* to adapt to new hosts and environments [76].

### Superantigens (SAgs)

SAgs, originally called staphylococcal enterotoxins, were named for causing food poisoning symptoms like vomiting and diarrhea [77]. However, as newer toxins in this group lacked emetic properties, the International Nomenclature Committee redefined the term in 2004 [6].

To date, 26 distinct SAgs have been identified in *S. aureus*. These include the toxic shock syndrome toxin (TSST-1), 11 staphylococcal enterotoxins (SEA–SEE, SEG–SEI, SER–SET), and 14 enterotoxin-like proteins (SElJ–SElQ, SElU–SElZ). While staphylococcal enterotoxins are known for their emetic activity, the enterotoxin-like (SEl) proteins either lack emetic properties in primate models or remain untested for such activity [78].

**Fig. 3:**
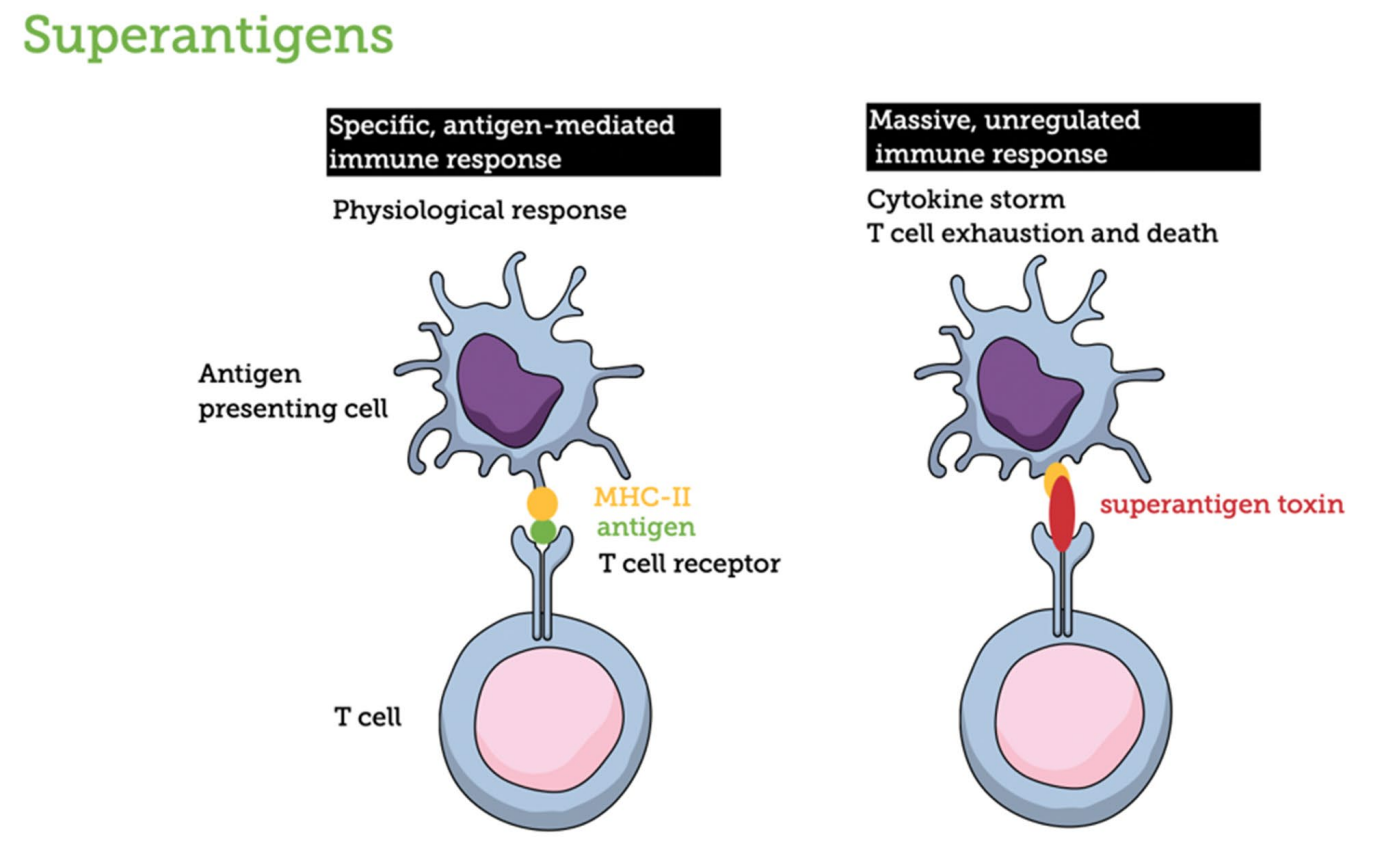
General principles of Superantigens mechanism. Superantigens can directly bind MHC class II molecules to T-cell receptors, triggering a constitutive and non-specific activation of T cells independent of specific antigens. This leads to a chain reaction resulting in the so-called cytokine storm and the massive activation of T cells, which ultimately undergo cell death

Most SAg genes are located on mobile genetic elements, including phages, pathogenicity islands, and plasmids, facilitating their transfer among *S. aureus* isolates through horizontal gene transfer. SAgs are the most potent T cell activators, capable of inducing massive immune responses at extremely low concentrations, even in the picomolar or femtomolar range [79]. Unlike conventional antigens, which require antigen-presenting cells such as dendritic cells, B cells, and macrophages to engulf, process, and present peptides on MHC-II molecules to CD4 + T cells, SAgs bypass these physiological pathways. Normally, CD4 + T cells recognize antigen-MHC-II complexes via the specific interaction of their T cell receptor α and β chains, leading to selective activation, clonal expansion, cytokine secretion, and support for B cells. However, SAgs disrupt this specificity by directly binding to T cell receptors and MHC-II molecules outside the peptide binding regions, activating up to 20% of all T cells regardless of antigen specificity. This indiscriminate activation triggers extensive T cell proliferation and the release of many proinflammatory cytokines, including IL-2, TNF-α, and IFN-γ. This cytokine storm causes severe symptoms, including fever, rash, vomiting, diarrhea, hypotension, and potentially multiple organ failure. Following this hyperactivation, T cells may enter a state of exhaustion, become unresponsive, or undergo cell death. On the antigen-presenting cell side, SAgs can induce varied responses depending on the cell type. For instance, in monocytes, SAgs can activate pathways via MHC-II dimerization or CD40 signaling, leading to the secretion of inflammatory cytokines like TNF-α, IL-1β, and IL-6 [8] (Fig. 3).

Besides their role in food poisoning, SAgs can trigger toxic shock syndrome (TSS) [80]. TSS is a severe and potentially life-threatening condition characterized by high fever, a widespread erythematous rash, low blood pressure, involvement of three or more organ systems, and skin desquamation occurring one to two weeks after onset—unless the illness proves fatal before this stage [81, 82]. These features are outlined in previous reviews, as well as specific diagnostic criteria and an extended description of the strains involved. Historically, it has been linked to both menstrual toxic shock syndrome (mTSS), first described in the 1980s, and non-menstrual cases, which have been reported during various outbreaks in the past century. Non-menstrual TSS can develop from any primary staphylococcal infection or colonization by a toxin-producing strain of *S. aureus* (including MRSA). It may occur following skin or mucous membrane disruption in connection with abscesses, burns, or surgical procedures [83, 84]. However, in many cases, no definitive source of infection is identified. Notably, subsets such as post-influenza illness, red syndrome, purpura fulminans, extreme pyrexia, and other soft tissue infections have been documented. Interestingly, in mTSS cases, TSST-1 is consistently produced, while α-hemolysin activity is almost universally absent. These cases also exhibit distinct biochemical and microbiological characteristics: for example, almost all mTSS isolates are methicillin-susceptible, and methicillin-resistant isolates lack gold pigmentation, appearing white instead. From an epidemiological perspective, studies on community-acquired MSSA and MRSA have shown an overall TSST-1 prevalence of 6% in the US [85]. Still, clonal epidemics may occur [86]. Of note, from a diagnostic standpoint, blood cultures are positive in fewer than 5% of cases of staphylococcal TSS. The clinical features of menstrual and non-menstrual TSS are generally similar. Menstrual TSS typically begins during menstruation in 95% of cases, while non-menstrual TSS is often acquired nosocomially, frequently after prior antibiotic treatment. Fever and rash are common early symptoms, with non-menstrual TSS more often involving central nervous system manifestations and renal complications. Non-staphylococcal enterotoxin A and non-TSST-1 superantigens appear to have higher neurotoxic potential. Post-operative non-menstrual TSS usually develops within 48 h of surgery, often without clear evidence of a surgical site infection. Symptoms progress rapidly, with multi-organ failure occurring within 8–12 h. While menstrual TSS can recur, non-menstrual TSS recurrences are rare. Non-menstrual TSS should be considered in cases of shock linked to suspected or confirmed SA infection. A thorough search for the source of the infection is crucial. Necrotizing fasciitis or myositis requires urgent and aggressive surgical debridement. Surgical wounds should be considered potential infection sites, even without clear signs. Infected wounds must be reopened, thoroughly debrided, and any packs or devices removed. For women, a vaginal examination should be performed [85, 86].

On the other hand, staphylococcal enterotoxins may lead to staphylococcal food poisoning caused by consuming foods containing enough preformed enterotoxins. Symptoms, which appear rapidly (2–8 h), include nausea, severe vomiting, abdominal cramps, and sometimes diarrhea. The condition is usually self-limiting, resolving within 24–48 h, though severe cases may require hospitalization. Food handlers carrying enterotoxin-producing *S. aureus* on their hands or in their nasal passages are the primary source of contamination. *S. aureus* is also found in food animals, particularly dairy cattle, sheep, and goats with subclinical mastitis, which can contaminate milk. Air, dust, and food contact surfaces can also act as vehicles. Commonly implicated foods include meat, poultry, egg, milk, dairy, salads, and baked goods. Staphylococcal food poisoning is widespread but likely underreported due to misdiagnosis, unreported minor outbreaks, and issues with sample collection or laboratory testing. Controlling this disease is essential for social and economic reasons [87].

#### Production of superantigens in various *S. aureus* strains

The distribution of superantigens in *S. aureus* is highly variable. It depends on the strain’s genetic background, its geographic origin, and the clinical context in which it is isolated. Superantigen genes are often located on mobile genetic elements, such as pathogenicity islands, phages, plasmids, or transposons, facilitating their horizontal transfer between strains. TSST-1 is predominantly associated with certain clonal lineages, such as those implicated in menstrual and non-menstrual TSS and is particularly common in strains isolated from healthcare environments [81, 82]. Enterotoxins, including SEA, SEB, SEC, and others, are widespread among *S. aureus* strains and are most linked to foodborne outbreaks [87, 88]. SEA and SEB, for instance, are frequently found in strains that contaminate food products, while SEC is often associated with mastitis in animals and zoonotic transmission to humans [89].

Some strains can harbor multiple superantigen genes, enhancing their virulence potential, particularly in systemic infections [90]. The presence of superantigens also varies geographically, as certain clonal lineages with specific superantigen profiles predominate in different regions. The variability in superantigen distribution reflects the evolutionary adaptability of *S. aureus* and its ability to exploit mobile genetic elements to expand its virulence arsenal across diverse ecological niches [91].

## Clinical implications and future therapeutic targets

Toxins are crucial to the pathology of *S. aureus* infections, enhancing its ability to cause disease by damaging tissues, disrupting immunity, and aiding infection spread. Key virulence factors include cytolysins, superantigens, and exfoliative toxins. Cytolysins, such as α- and γ-hemolysins, compromise cellular membranes, leading to tissue necrosis and immune cell lysis. Superantigens, notably TSST-1 and staphylococcal enterotoxins, overstimulate T cells, resulting in cytokine storm and potential organ failure. Exfoliative toxins target desmoglein-1 in the epidermis, causing epidermal separation and conditions such as SSSS [64].

The production of these toxins differs between MSSA and MRSA due to their genetic backgrounds and the selective pressures they face in different environments (Table 2). Studies have shown that over 90% of MSSA and MRSA strains produce α-hemolysin [92]. In contrast, the presence of PVL differs significantly between strain types: it is detected in approximately 5% of MSSA strains [93], whereas it can be found in up to 60% of CA-MRSA strains, particularly in North America and Europe, where epidemic clones such as USA300/ST8 and ST80 predominate [94, 95]. When examining superantigen profiles, MSSA strains generally display greater diversity, with 60–80% reported to harbor genes encoding for staphylococcal enterotoxins such as SEA, SEB, and SEC, as well as TSST-1 [96]. Exfoliative toxins also appear to be more commonly associated with MSSA, which represents the principal reservoir of ETA and ETB, the toxins responsible for SSSS [61, 64, 97].

In a recent Greek clinical study, researchers assessed the impact of toxin gene presence on mortality outcomes. Among a cohort of 149 *S. aureus* strains, 21.5% carried the *lukS/lukF*-PV genes, while 9.4%, 78.7%, 2.7%, and 0.7% carried the *tst*, *fnbA*, *eta*, and *etb* genes, respectively. No significant differences were observed between toxin gene presence and mortality [98]. Overall, MSSA strains display a broader toxin repertoire, reflecting their genetic diversity and adaptation to community settings. In contrast, CA-MRSA, are more specialized, producing high levels of specific toxins, while HA-MRSA strains are primarily adapted to survival in healthcare environments with a toxin profile dominated by TSST-1 and PFTs, with reduced exfoliative toxin production, likely due to evolutionary pressures [99].

**Table 2:** Comparison of toxin type distribution in MSSA *versus* MRSA

Toxin Type	Toxin Name	MSSA (% of strains)	MRSA (% of strains)
Pore-Forming Toxins	Alpha-toxins	> 90%	> 90%
	PVL	5%	60% (CA-MRSA)
Exfoliative Toxins	ETA	80%	< 5%
	ETB	20–30%	< 5%
Superantigens	Enterotoxins (SEA, SEB, SEC)	60–80%	Varies by lineages, often lower
	TSST-1	20–30%	50% (HA-MRSA)

**Fig. 4:**
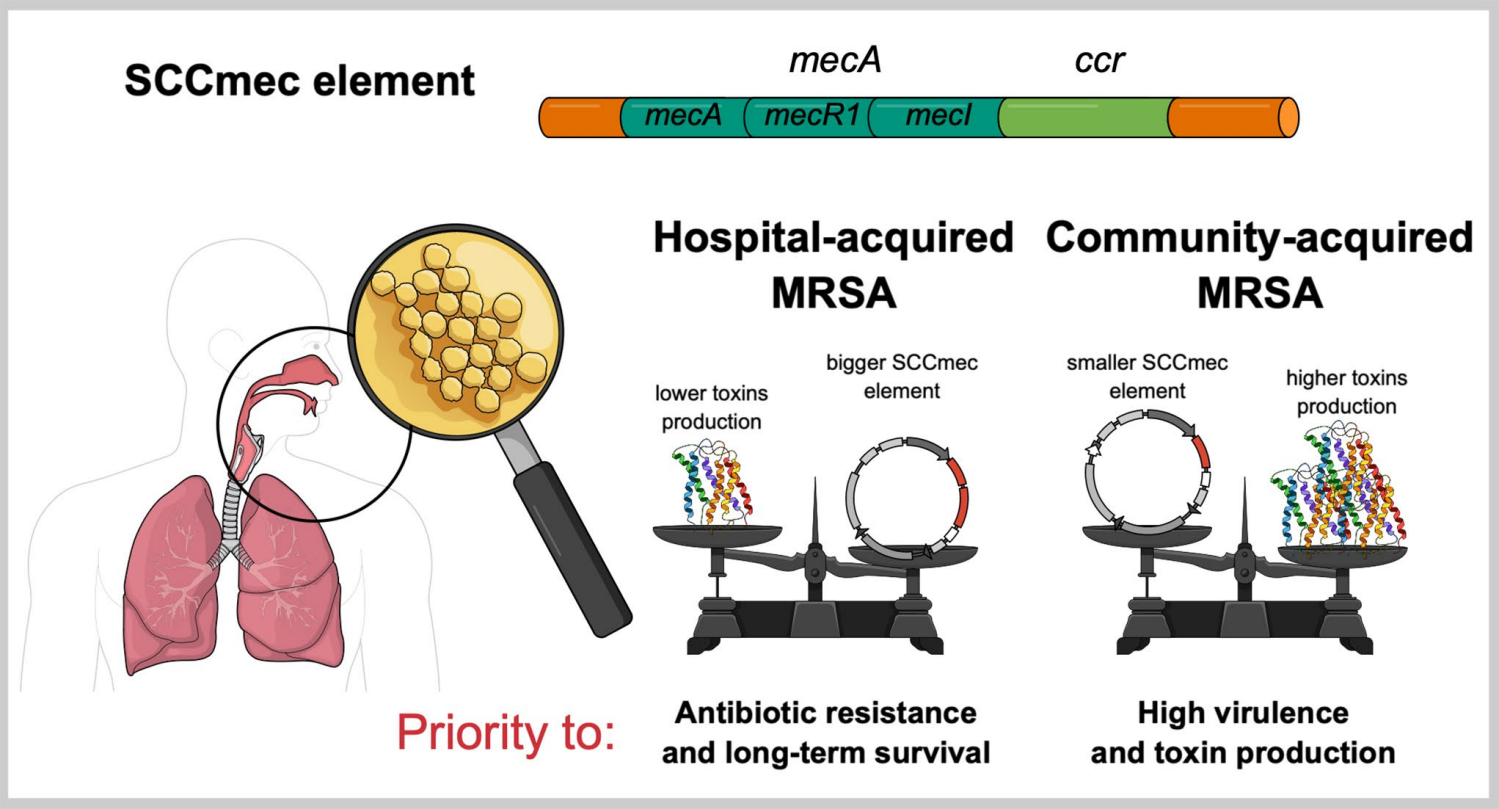
Relationship between SCC*mec* element and toxin production in HA-MRSA vs. CA-MRSA

An indirect relationship exists between toxin expression and the staphylococcal cassette chromosome *mec* (SCC*mec*)-types (Fig. 4). SCC*mec* is the mobile genetic element that confers methicillin resistance through the *mecA* gene, which encodes the low-affinity penicillin-binding protein PBP2a [100]. This element imposes evolutionary pressure on *S. aureus* strains, particularly in hospital settings with high antibiotic use. This pressure indirectly influences toxin production, as HA-MRSA strains often exhibit reduced toxin expression compared to CA-MRSA. This reflects a trade-off between survival and virulence, with HA-MRSA tending to prioritize antibiotic resistance and colonization over toxin production [101–103]. HA-MRSA strains typically carry large SCC*mec* elements, such as type II and III, and produce fewer toxins like PVL [101].

Although *SCCmec* does not directly encode toxins, it is often acquired with other mobile genetic elements, such as pathogenicity islands, phages, or plasmids, that may carry toxin genes (e.g., PVL, TSST-1, or enterotoxins) [101, 103].

SCC*mec* also indirectly modulates virulence through interactions with global regulatory systems, most notably the accessory gene regulator (*agr*) and the staphylococcal accessory regulator A (*sar*A). The *agr* system, a quorum-sensing system, regulates the expression of a wide array of virulence factors, including toxins, surface proteins,, and enzymes. It comprises two divergent transcripts: RNAII (encoding the AgrA, AgrC, AgrD and AgrB proteins) and RNAIII (the main effector RNA). AgrD, the autoinducing peptide, activates the AgrC-AgrA pathway once a threshold concentration is reached, leading to downstream gene regulation [104].

*sar*A, a DNA-binding protein, modulates a broad spectrum of virulence genes, either independently or in coordination with *agr*. While the exact interplay between SCC*mec*, *sar*A, and *agr* remains incompletely understood, it is plausible that the SCC*mec* influence on *agr* could indirectly affect *sar*A-mediated regulation as well, further impacting toxin production [105, 106]. In summary, the relationship between SCC*mec* and toxin expression is multifactorial, involving evolutionary pressures, regulatory interplay, and energy balance [102, 107].

Furthermore, the presence of SCC*mec* can alter the cell’s metabolic state, which in turn can influence *agr* activity, as *agr* is sensitive to environmental cues. These dynamics help explain the distinct virulence profiles observed in CA-MRSA versus HA-MRSA strains [101, 105].

**Fig. 5:**
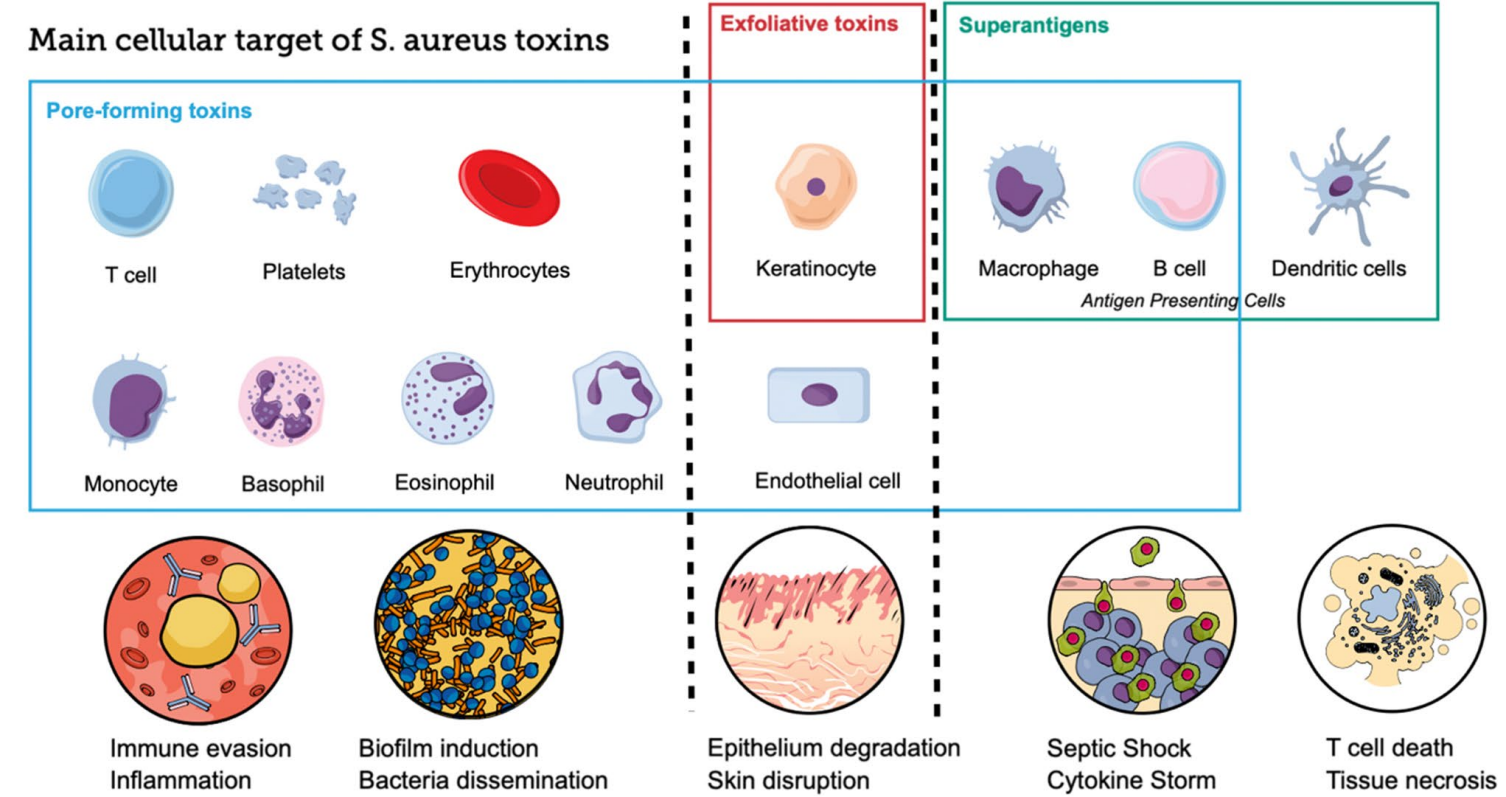
Main cell type targets for the different toxins are shown

Broadly, toxins represent a significant clinical challenge because they complicate treatment and recovery by targeting different cell types and exerting different actions (Fig. 5).

The interaction between the host and virulence factors is complex, and it remains unclear whether highly virulent or less virulent strains have an impact at different infection stages. Moreover, the relative contribution of individual toxins versus their synergistic action is still not fully understood. An important area of investigation involves identifying when *S. aureus* reduces toxin production [108].

Investigating *S. aureus* internalization and survival in host cells is crucial for addressing its infectivity and pathogenicity, laying the groundwork for future vaccine and therapeutic strategies.

Toxin-mediated damage is often rapid and severe, making timely intervention critical. While antibiotics are effective in killing the bacteria, they do not neutralize toxins already released, meaning the damage can continue even after the infection is “controlled”. Furthermore, toxins can exacerbate systemic complications, such as sepsis or toxic shock syndrome, increasing mortality rates.

Antibiotics, such as vancomycin and daptomycin, are the mainstay treatment for eliminating *S. aureus*, though they do not directly target the toxins themselves [109]. The use of adjunctive antibiotics targeting exotoxin production in *S. aureus* infections is common and recommended in numerous guidelines despite being supported by limited evidence. These recommendations are primarily based on the theoretical premise of toxin suppression. Consequently, clinicians are prone to add a second agent with the aim of inhibiting exotoxin release by *S. aureus*, specifically in necrotizing pneumonia, complicated SSTIs or severe *S. aureus* infections [110, 111]. Among these agents, clindamycin, a protein synthesis-inhibiting lincosamide, is frequently employed due to its well-established efficacy and favorable pharmacological profile. It is active during the stationary phase, lacks an inoculum effect, and is capable of suppressing penicillin-induced exotoxin production. Other anti staphylococcal antibiotics with protein synthesis-inhibiting properties, includes, linezolid, rifampin, aminoglycosides, and tetracyclines. Nevertheless, clindamycin remains a preferred choice because of its accessibility, cost-effectiveness, and consistent performance [110, 111]. More recently, an innovative protocol has been designed to evaluate the specific role of adding clindamycin to the standard of care in *S. aureus* bacteremia [112].

In addition to antibiotics, intravenous immunoglobulins (IVIG) have been used successfully to neutralize certain toxins, such as TSST-1, thereby mitigating the severity of TSS in severe cases [113]. Additionally, IVIG preparations have shown functional activity against *S. aureus* leukocidin LukAB.

In experimental models, IVIG has demonstrated multiple beneficial effects. In a murine peritonitis model, it significantly enhanced neutrophil-mediated MRSA killing at all tested concentrations, reduced hemolytic activity tenfold, and decreased bacterial coagulation ability by 50% [114]. Additional in vitro studies have shown that IVIG can effectively neutralize pore formation and the cytopathic effects induced by PVL and *S. aureus* culture supernatants. In a rabbit model, IVIG prophylaxis protected against necrotizing pneumonia caused by five distinct CA-MRSA epidemic strains and one HA-MRSA strain, likely through neutralization of α-hemolysin and PVL. Despite these promising data, clinical use of IVIG remains limited, owing to inconclusive evidence and uncertainties regarding timing and patient selection [115, 116].

Monoclonal antibodies (mAbs) are also being investigated for their ability to neutralize specific *S. aureus* toxins, offering promise for managing resistant infections. For instance, a study investigated mAbs against α-hemolysin and clumping factor A, demonstrating enhanced protection in a lethal bacteremia model [117]. Another recent study identified a human anti-α-hemolysin mAb that effectively neutralize the toxin’s activity and protect against infection in a murine model [100]. Further studies have assessed combination of mAbs targeting -α-hemolysin/PVL/ γ-hemolysin and clumping factor A. These were tested in rabbit models of septic shock and necrotizing pneumonia caused by CA-MRSA USA300, with encouraging results [118–120]. Altogether, these findings support the therapeutic potential of mAb-based strategies in mitigatingtoxin-mediated damage during *S. aureus* infections.

Anti-inflammatory therapies, particularly corticosteroids, represent another adjunctive option aimed at curbing the excessive immune response triggered by superantigens, such as cytokine storms. However, their use in *S. aureus* infections remains controversial. While corticosteroids are effective in reducing inflammation, they also suppress immune function, potentially increasing the risk of uncontrolled bacterial growth and masking secondary infections [121, 122].

Nevertheless, in selected cases, such as staphylococcal TSS, some studies suggest that adding corticosteroids to antibiotic therapy could improve clinical outcomes by reducing systemic inflammation [123]. However, the evidence is limited, and their use must be carefully weighed on a case-by-case basis.

Surgical drainage or debridement is often required in cases of deep-seated infections, abscesses, or device-associated infections. In particular, removing infected prosthetic devices, such as catheters, heart valves, or joint implants, is often essential in managing toxin-producing S. aureus infections [86, 124, 125]. In toxin-mediated conditions like TSS or necrotizing fasciitis, timely and aggressive surgical intervention is crucial to control the source of toxin production and prevent systemic complications [126, 127].

A variety of inhibitory compounds are currently under investigation for their potential to neutralize *S. aureus* toxins or disrupt their production. These include small molecules, peptides, engineered T cell receptors, and RNA-based therapeutics.

Recent experimental evidence has shown that small molecules from the quinoxalinedione class can selectively inhibit *S. aureus* α-hemolysin by blocking pore formation and reducing tissue damage. In murine models of staphylococcal pneumonia, these compounds improved survival even without antibiotics, suggesting a “path blocker” therapeutic approach focused on neutralizing virulence rather than bacterial growth [128].

Small molecules such as apicidin, a histone deacetylase inhibitor, interfere with the regulatory systems that control toxin production. Similarly, hydroquinone and its derivatives have been shown to suppress the *agr* system, therefore downregulating toxin expression [129].

Peptides, including engineered antimicrobial peptides and toxin-neutralizing antibodies, act by directly binding to toxins or disrupting their activity, thereby neutralizing their cytopathic effects [130, 131].

Soluble engineered T cell receptors (TCRs) represent another promising strategy against *S. aureus* superantigens by competitively blocking their interaction with MHC class II molecules and native TCRs, thus preventing aberrant T cell activation and cytokine storm [132]. In rabbit models of MRSA-induced necrotizing pneumonia and endocarditis, an anti-SEC TCR significantly reduced bacterial burden and improved survival [133]. Similar results were obtained with engineered TCRs targeting TSST-1 and SEB, both in vitro and in vivo [96].

RNA-based approaches, such as antisense RNA molecules, have been developed to target the genetic sequences encoding specific toxins, thereby preventing their expression [2].

Together, these compounds represent a range of strategies to mitigate the impact of *S. aureus* toxins, although further research is required to optimize their clinical use.

In parallel, vaccines targeting specific toxins, such as TSST-1 and enterotoxins, are also in development and may offer a promising preventative strategy. Several candidate vaccines are based on detoxified toxins variants or recombinant protein fragments, designed to stimulate the production of neutralizing antibodies [134–136]. For example, vaccines targeting α-toxin have shown promise in preclinical and early-phase clinical trials, reducing the severity of infections in animal models. Research is also underway to develop multivalent vaccines that simultaneously target multiple toxins, thereby broadening protection acrossdifferent *S. aureus* strains and clinical scenarios [136].Despite promising advances, developing effective vaccines against *S. aureus* toxins has proven challenging due to the diversity of the toxins, the complexity of their interactions with the host immune system, and the ability of the bacterium to evade immune defenses.

## Conclusion

In conclusion, by combining antimicrobial therapies with approaches that neutralize toxins and modulate the immune response, clinicians can improve outcomes in patients affected by *S. aureus* infections. Further research into these strategies will be critical for addressing the challenges posed by this versatile and formidable human pathogen.

## Data Availability

No datasets were generated or analysed during the current study.
